# Using clinical and genetic risk factors for risk prediction of 8 cancers in the UK Biobank

**DOI:** 10.1093/jncics/pkae008

**Published:** 2024-02-14

**Authors:** Jiaqi Hu, Yixuan Ye, Geyu Zhou, Hongyu Zhao

**Affiliations:** Department of Chronic Disease Epidemiology, Yale School of Public Health, New Haven, CT, USA; Program of Computational Biology and Bioinformatics, Yale University, New Haven, CT, USA; Program of Computational Biology and Bioinformatics, Yale University, New Haven, CT, USA; Program of Computational Biology and Bioinformatics, Yale University, New Haven, CT, USA; Department of Biostatistics, Yale School of Public Health, New Haven, CT, USA

## Abstract

**Background:**

Models with polygenic risk scores and clinical factors to predict risk of different cancers have been developed, but these models have been limited by the polygenic risk score–derivation methods and the incomplete selection of clinical variables.

**Methods:**

We used UK Biobank to train the best polygenic risk scores for 8 cancers (bladder, breast, colorectal, kidney, lung, ovarian, pancreatic, and prostate cancers) and select relevant clinical variables from 733 baseline traits through extreme gradient boosting (XGBoost). Combining polygenic risk scores and clinical variables, we developed Cox proportional hazards models for risk prediction in these cancers.

**Results:**

Our models achieved high prediction accuracy for 8 cancers, with areas under the curve ranging from 0.618 (95% confidence interval = 0.581 to 0.655) for ovarian cancer to 0.831 (95% confidence interval = 0.817 to 0.845) for lung cancer. Additionally, our models could identify individuals at a high risk for developing cancer. For example, the risk of breast cancer for individuals in the top 5% score quantile was nearly 13 times greater than for individuals in the lowest 10%. Furthermore, we observed a higher proportion of individuals with high polygenic risk scores in the early-onset group but a higher proportion of individuals at high clinical risk in the late-onset group.

**Conclusion:**

Our models demonstrated the potential to predict cancer risk and identify high-risk individuals with great generalizability to different cancers. Our findings suggested that the polygenic risk score model is more predictive for the cancer risk of early-onset patients than for late-onset patients, while the clinical risk model is more predictive for late-onset patients. Meanwhile, combining polygenic risk scores and clinical risk factors has overall better predictive performance than using polygenic risk scores or clinical risk factors alone.

Cancer is a widespread disease that accounts for millions of deaths each year. It is estimated that nearly 10 million deaths were caused by cancer in 2020 (1). The etiology of cancer can be attributed to genetic and nongenetic factors. In recent years, large-scale genome-wide association studies (GWASs) have identified thousands of single-nucleotide variations (SNVs—formerly SNPs) associated with cancer (2-7). For example, a recent GWAS combining 100 204 colorectal cancer (CRC) cases and 154 587 controls of European and East Asian ancestry through meta-analysis successfully identified 205 independent SNVs strongly associated with CRC (4), demonstrating the role of genetic factors in cancer development. Nongenetic factors, such as increased age (8) and smoking (9) are widely known as general risk factors of cancer. Additionally, studies have identified risk factors specific to certain cancers. For instance, reproductive factors, such as the use of exogenous hormones, have been associated with an increased risk of breast cancer (10).

Polygenic risk scores have been proposed to accurately predict the risk of developing cancer by aggregating the genetic effects of SNVs (11-13). The comprehensive analysis of polygenic risk scores for 8 types of cancer found that people with the top percentile of polygenic risk scores had a higher risk of developing cancer (11), demonstrating that polygenic risk scores could accurately predict the risk of cancer. Combining cancer-specific polygenic risk scores with demographic variables, family history, and other modifiable variables could further improve risk prediction and identify high-risk individuals (12). The interaction between genetic and clinical risk factors may contribute to cancer risk, as well. For example, age of onset was found to interact with genetic risk, and patients with early-onset breast cancer had a higher proportion of high polygenic risk scores, while patients with early-onset prostate cancer had a higher proportion of high clinical risk (13).

Previous studies have demonstrated the feasibility of integrating genetic and clinical risk factors for predicting cancer risk, but these studies have had several limitations. First, previous polygenic risk scores were developed using preselected SNVs, which relied strongly on prior knowledge and potentially lacked power because of the limited number of SNVs. Second, previous research was restricted to a limited number of methods for polygenic risk score construction, which could be further improved by employing methods that incorporate information such as linkage disequilibrium (LD) and functional annotation. Third, previous research relied on various approaches to selecting clinical risk factors, highlighting the need for an objective and generalizable approach in choosing clinical variables that effectively capture cancer risk. Finally, the impact of the age of onset on cancer risk is controversial and requires more evidence.

To fulfill the current research gap, this study integrated the best-performed polygenic risk score from various methods and clinical risk factors, selected by Extreme Gradient Boosting (XGBoost), a machine learning algorithm, into risk-prediction models for 8 cancers (bladder, breast, colorectal, kidney, lung, ovarian, pancreatic, and prostate cancers) using the UK Biobank dataset. We aimed to develop accurate risk-prediction models for specific cancers. Furthermore, we explored the interaction between the age of cancer onset and genetic and clinical risk factors to determine the optimal model in practice for patients with varying ages of onset. Our findings indicated that polygenic risk scores can accurately predict risk 8 cancers, adding clinical variables selected by XGBoost could further improve the prediction, and the models could be interpreted with clinical and biological implications. Analysis of the age of onset showed that for patients with early-onset disease, polygenic risk score models were more accurate, while models with clinical risk factors were more suitable for patients with late-onset disease.

## Methods

### Study population

Our participants were from the UK Biobank, a large-scale, prospective cohort study that started in 2006 and recruited more than 500 000 middle-aged individuals across the United Kingdom (14). Participants’ medical information was obtained through linkage to Hospital Episode Statistics (HES), national death registries, and cancer registries. More specifically, HES patients were diagnosed based on both *International Classification of Diseases, Ninth Revision* and *International Statistical Classification of Diseases, Tenth Revision*, and the surgical procedures were recorded in the Office of Population Censuses and Surveys Classification of Interventions and Procedures (Population, Census, and Census Office: Classification of Interventions and Procedures, version 4). HES data from April 1994 to March 2017 were currently available for patients admitted to National Health System hospitals in England and Scotland. Death registries covered all deaths in the United Kingdom up to February 2016, and registrations of cancer were from the 1960s to September 2015. For this study, to decrease the population stratification effects, only White British patients were included in the analysis.

We used phase III genotype data released by the UK Biobank where the participants underwent genotyping with 1 of 2 closely related Affymetrix microarrays (UK BiLEVE Axiom Array or UK Biobank Axiom Array; Applied Biosystems/Thermo Fisher Scientific, Waltham, MA) for approximately 820 000 variants. Additional genotypes were imputed centrally using the 1000 Genomes and the Haplotype Reference Consortium reference panels, yielding approximately 93 million variants for each individual. We restricted the analysis to 2 994 054 autosomal variants with imputation quality scores greater than 0.3, Hardy-Weinberg *P* larger than 1e-5, a minor allele frequency greater than 0.05, and a genotyping missing rate less than 0.01.

The patients with cancer in the UK Biobank were diagnosed through the integration of self-report and HES data. Specific definition codes can be found in Supplementary Table 1 (available online). To exclude the possibility of reverse-causality, patients with preexisting diagnoses for the same type of cancer were excluded. The follow-up time started on the recruitment day and ended on the date of cancer incidence for cases and the date of death or the end of the study, whichever came first, for controls.

### Polygenic risk score derivation

Summary statistics from publicly available, large-scale GWASs were used to develop the polygenic risk scores for 8 cancers. The summary-level data did not include the UK Biobank summary statistics in the meta-analysis. Details regarding sample sizes and heritability estimates can be found in Supplementary Table 2 (available online).

To enhance the accuracy of polygenic risk scores in risk prediction, we employed 5 methods: Pruning and Thresholding (P + T) (15), PRS-CS (16), LDPred2 (17), AnnoPred (18), and SDPR (19). We used 503 European patients from the 1000 Genomes project phase III (20) as the external LD reference panel. The method with the best prediction performance was chosen for each cancer type.

### Clinical variable selection

To develop a clinical prediction model for cancer risk, we aggregated information for 733 traits measured during the baseline visit in the UK Biobank. The traits analyzed and the corresponding field identifiers in the UK Biobank are provided in Supplementary Table 3 (available online). We ran XGBoost (21) to assess the relative contributions of traits to different cancers. XGBoost is a scalable, gradient-boosting tree algorithm specifically designed for handling large-scale sparse data, making it well suited to analyzing datasets such as the UK Biobank’s. The parameters of the algorithm were tuned individually for each cancer type for optimal prediction performance with the highest accuracy. The feature importance of each trait was retained for subsequent analysis.

Following XGBoost, we kept traits that had non-zero feature importance and missingness rates below 5%. The remaining traits were imputed using the mean for continuous variables and the reference value for categorical variables. The imputation methods were decided based on the simplicity and unbiased estimates. Furthermore, a forward selection process was conducted to determine the number of traits included in the model. The traits were first ranked by feature importance in descending order. The process began with a model incorporating the top-ranked trait (the trait with the largest feature importance), followed by the incremental addition of 1 trait at a time. In each round, the prediction performance of the model was assessed through the area under the curve (AUC). The number of traits included was decided by an optimal trade-off between a small number of traits and a good prediction performance.

### Statistical analysis

The data were partitioned into training and testing sets using a 9:1 ratio, and the best polygenic risk scores and clinical variables were chosen in the training samples. For subsequent analyses, to maximize power, we combined the training and testing sets (Supplementary Figure 1, available online). This study used the Cox proportional hazards model. The prediction performance was measured by AUC and hazard ratio through 5-fold cross-validation. The covariates in this study were sex (if applicable), age at recruitment, and top-10 principal components derived using 407 219 unrelated, UK Biobank patients and 147 604 pruned SNVs after quality control from the previous study (22).

Age has been recognized as an important factor in cancer development. To explore the interaction between the age of onset and genetic and clinical risk, we divided the patients with cancer into early- and late-onset groups with different cut points. The specific cutoff for the age of onset was determined by searching the American Cancer Society (23). We further defined the high–polygenic-risk-score and high–clinical-risk groups as the top 10% quantile of polygenic risk score and clinical risk score, respectively. The proportions of high–polygenic-risk-score and high–clinical-risk individuals in the early-onset and late-onset groups were compared to explore the impact of age of onset.

The significance level was set at .05, and the Bonferroni correction was applied to adjust for multiple comparisons. XGBoost was performed in Python, version 3.9.7, and the polygenic risk scores were calculated using PLINK, versions 1.90 (24) and 2.0 (15). Other analyses were done in R, version 4.2.0 (R Foundation for Statistical Computing, Vienna, Austria).

### Sensitivity analysis

In XGBoost, a logistic regression model was used for risk prediction. In subsequent analysis, however, we employed the Cox proportional hazards model, resulting in a potential inconsistency. To assess the impact, a sensitivity analysis was conducted by fitting a logistic regression model on the combined training and testing samples for polygenic risk score selections, the forward selection of clinical variables, and model prediction performance assessment.

Additionally, due to the limited sample size for some cancer types, we combined the training and testing samples for the primary analysis, which may overestimate the prediction performance of the models. To validate the approach, a similar analysis was repeated based on testing samples, and the results were compared with those using the combined data.

In addition, we calibrated the model using testing samples. We first trained the model using 50% of the samples and predicted the 5-year survival for the rest of the samples. The model calibration was assessed using the Hosmer-Lemeshow goodness-of-fit statistic, modified for time-to-event outcomes (25), and we plotted the calibration lines following the methodology in a previous paper (12).

## Results

### Population characteristics

The number of individuals analyzed varied for each cancer type, with the number of patients ranging from 1093 for ovarian cancer to 8889 for prostate cancer. The number of controls was 405 180 for bladder cancer, 204 014 for breast cancer, 400 850 for CRC, 406 794 for kidney cancer, 404 219 for lung cancer, 219 097 for ovarian cancer, 407 388 for pancreatic cancer, and 176 296 for prostate cancer. Table 1 presents a summary of the demographic characteristics of both the general population and the patients with cancer. The patients with cancer showed an older average age and higher body mass index than did the general population.

**Table 1.** Summary of demographic variables across 8 cancer types and the general population

	All (N = 409 532)	Bladder (n = 2588)	Breast (n = 8407)	Colorectal (n = 5607)	Kidney (n = 1548)	Lung (n = 4263)	Ovarian (n = 1093)	Pancreatic (n = 1402)	Prostate (n = 8889)
Sex (No. male), %	45.95 (188 184)	75.62 (1957)	0 (0)	56.55 (3171)	64.02 (991)	51.91 (2213)	0 (0)	54.56 (765)	100 (8889)
Age at recruitment, mean (SD), y	56.91 (8)	62.22 (5.78)	57.84 (7.61)	60.91 (6.52)	60.76 (6.38)	61.91 (5.73)	59.45 (7.32)	61.64 (6.12)	61.74 (5.58)
Townsend deprivation index, mean (SD)	‒1.56 (2.94)	‒1.48 (3)	‒1.62 (2.85)	‒1.6 (2.97)	‒1.52 (3.04)	‒0.3 (3.49)	‒1.62 (2.88)	‒1.38 (3.09)	‒1.85 (2.82)
Body mass index, mean (SD), kg/m^2^	27.42 (4.76)	28.11 (4.5)	27.46 (5.07)	27.98 (4.69)	28.89 (4.99)	27.51 (4.74)	27.33 (5.01)	28.24 (4.91)	27.66 (3.83)
Systolic blood pressure, mean (SD), mm Hg	140.2 (19.66)	145.35 (19.55)	139.37 (20.41)	144.32 (20)	145.59 (19.7)	143.47 (19.98)	139.53 (19.51)	144.37 (19.86)	145.63 (18.62)
Diastolic blood pressure, mean (SD), mm Hg	82.25 (10.66)	83.2 (10.62)	81.32 (10.54)	83.07 (10.63)	83.83 (10.71)	81.59 (10.59)	80.74 (10.01)	82.76 (10.89)	84.11 (10.38)

### Selection of polygenic risk score methods

Five methods (P + T, PRS-CS, LDpred2, AnnoPred, and SDPR) were used to construct polygenic risk scores for 8 cancer types. The method that achieved the highest AUC for predicting respective cancers in the training samples was chosen (Table 2). Specifically, LDPred2 demonstrated the highest AUC values for bladder cancer (0.563), CRC (0.567), and lung cancer (0.587). For breast and prostate cancers, SDPR outperformed the other methods, with AUC values of 0.656 and 0.681, respectively. AnnoPred showed the highest accuracy among the 5 methods for kidney cancer (0.547), ovarian cancer (0.536), and pancreatic cancer (0.549). Notably, breast and prostate cancer, the 2 cancer types with the largest number of cases in the summary statistics (Supplementary Table 2, available online), displayed higher AUC values than the other cancers.

**Table 2.** The highest area under the curve values of 5 polygenic risk score methods for 8 cancer types in training samples

	P + T	PRS-CS	LDpred2	AnnoPred	SDPR
Bladder	0.5614	0.536	0.5632	0.556	0.556
Breast	0.5876	0.6514	0.654	0.6112	0.656
Colorectal	0.555	0.5456	0.5672	0.564	0.5642
Kidney	0.5426	0.5234	0.54	0.547	0.5216
Lung	0.5708	0.5774	0.5866	0.575	0.5826
Ovarian	0.517	0.5228	0.5174	0.5364	0.5262
Pancreatic	0.5358	0.535	0.5426	0.5488	0.536
Prostate	0.66	0.6802	0.6684	0.66	0.6806

### Clinical variables

**Figure 1.**
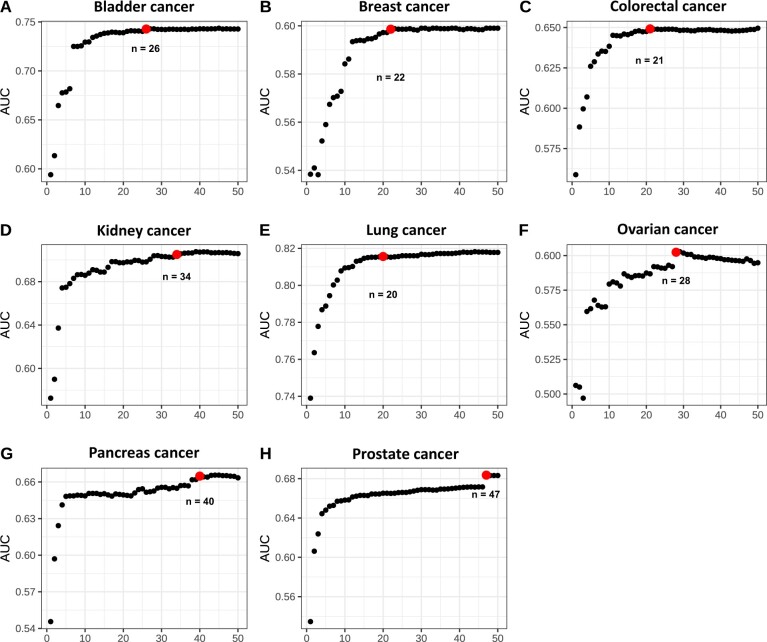
**AUCs of models with an accruing number of traits across 8 cancer types**. The Cox proportional hazards models were fitted and the prediction performance was evaluated, with an increasing number of clinical traits ranked by feature importance. The relationship between AUC and the number of clinical traits included is shown. The elbow points, which represent the optimal trade-off between AUC and the number of traits, are marked and labeled accordingly. The final number of traits ranged from 20 for lung cancer (1-e) to 47 for prostate cancer (1-h). AUC = area under the curve.

Cancer-specific XGBoost models were trained; parameters are shown in Supplementary Table 4 (available online). The set of traits with non-zero feature importance that XGBoost selected was pruned based on a missingness rate less than 5%; to further select traits that best predict cancer risk, we computed the AUC values for prediction models with traits of top feature importance. The number of clinical variables was determined by identifying the elbow point that balanced the number of variables and the AUC (Figure 1). Finally, 26 traits for bladder cancer, 22 traits for breast cancer, 21 traits for CRC, 34 traits for kidney cancer, 20 traits for lung cancer, 28 traits for ovarian cancer, 40 traits for pancreatic cancer, and 47 traits for prostate cancer were included. Supplementary Table 5 (available online) shows the traits selected for the 8 cancer types.

**Figure 2.**
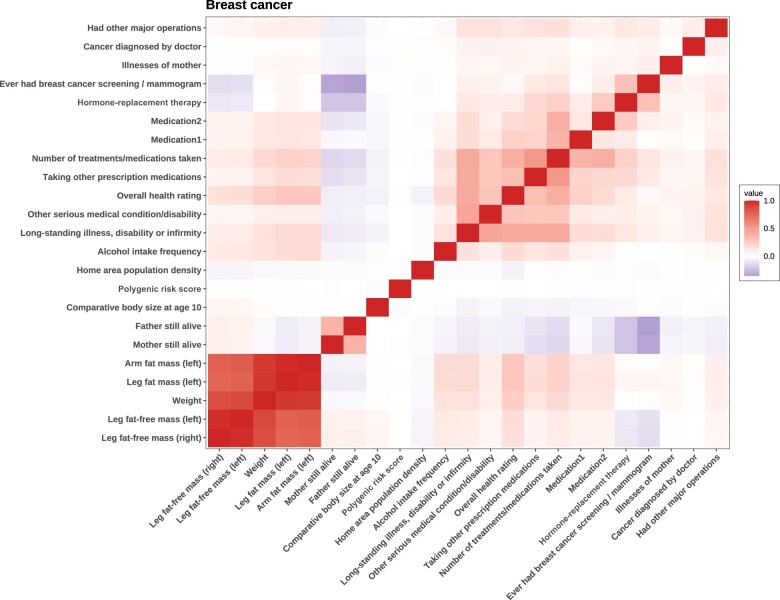
**Correlations between traits and polygenic risk score for breast cancer**. Correlations among variables are calculated, but the correlation between polygenic risk scores and clinical variables was too small and could be neglected, suggesting that the polygenic risk scores and clinical factors captured distinct components for the risk of breast cancer. Notably, we identified several clusters of highly correlated clinical traits, such as the obesity cluster and the overall health condition cluster. These patterns were consistently observed across other cancer types, indicating similarities in clinical risk factors. Slashes between words in labels represent ‘or’.

Correlations between clinical traits and polygenic risk scores for breast cancer are shown in Figure 2. We observed distinct clusters of traits as obesity-related traits (fat mass, fat-free mass, and weight), overall-health ratings (medicines taken and long-standing illness), and reproductive factors (mammogram and hormone-replacement therapy). Additionally, the polygenic risk scores for breast cancer showed only modest correlations with clinical traits. Similar patterns could be observed for the other 7 cancer types as well (Supplementary Figure 2, available online).

### Risk-prediction model

**Figure 3.**
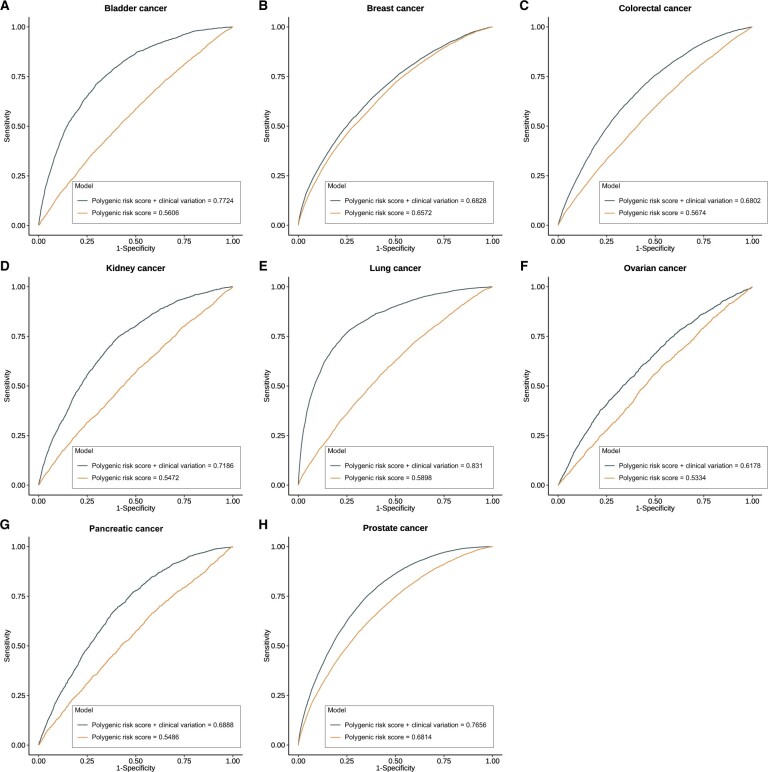
**Receiver operating characteristic curves of polygenic risk scores only and integration of polygenic risk scores and clinical variables for 8 cancer types**. We used Cox proportional hazards models to predict risk for 8 cancer types, with polygenic risk scores only or the combination of polygenic risk scores and clinical variables. The polygenic risk scores showed moderate prediction performance, with area under the curve values ranging from 0.53 for ovarian cancer to 0.68 for prostate cancer. Furthermore, integrating polygenic risk scores and clinical variables improved the prediction accuracy by 3.9% to 40.9%. The most substantial improvement was found for lung cancer, with an area under the curve of 0.83 for the combined model and 0.59 for the polygenic risk score model.

Prediction models incorporating both polygenic risk scores and selected clinical variables were constructed for each cancer type. We checked the prediction performance through receiver operating characteristic curves and AUC values. The polygenic risk scores alone demonstrated moderate to strong predictive accuracy for 8 cancer types, with AUC values ranging from 0.533 for ovarian cancer to 0.681 for prostate cancer (Figure 3). Adding clinical variables could statistically significantly improve the AUC for bladder cancer, CRC, kidney cancer, lung cancer, and pancreatic cancer (Figure 3). More specifically, the AUC values of models with polygenic risk scores only and both polygenic risk scores and clinical factors were 0.56 vs 0.77, 0.57 vs 0.68, 0.55 vs 0.72, 0.59 vs 0.83, and 0.55 vs 0.69 for these cancers, respectively. For breast, ovarian, and prostate cancers, respectively, models with both clinical factors and genetic risk showed slightly higher prediction accuracy, with AUC values of 0.68, 0.62, and 0.77, respectively, compared with 0.66, 0.53, and 0.68, respectively, for models with polygenic risk scores only. Furthermore, we compared our models with well-established risk-prediction models for breast and lung cancers. For breast cancer, we used a subset of the International Breast Cancer Intervention Study (IBIS) model (26) (6 variables unavailable, details in Supplementary Table 6, available online). The partial IBIS model yielded an overall AUC of 0.67, slightly lower than our model (0.68, 95% confidence interval [CI] = 0.67 to 0.7), with no statistically significant difference identified. For lung cancer, the National Cancer Institute Prostate Lung Colorectal and Ovarian Cancer Screening Trial (PLCO) model (27), excluding 2 unavailable variables, was used, along with sex, top-10 principal components, and polygenic risk scores. The AUC of the modified PLCO model (0.82, 95% CI = 0.81 to 0.83) was lower than our model (0.83, 95% CI = 0.82 to 0.84) but not statistically significant.

**Figure 4.**
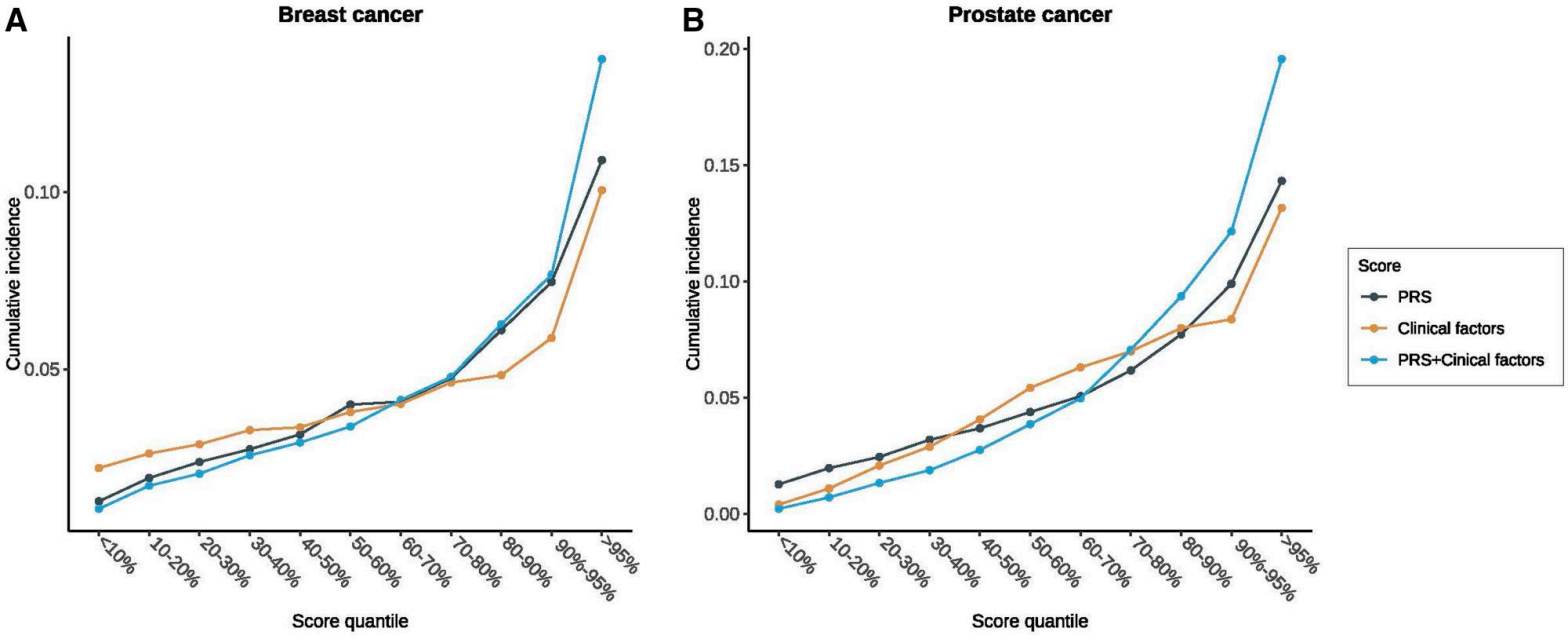
**Cumulative incidence of breast and prostate cancer in quantiles of 3 scores**. We divided the polygenic risk score, clinical factor risk score, and combined score into 11 quantiles and calculated the cumulative incidence of cancer in each quantile. All 3 scores were able to differentiate between individuals at high risk and individuals at low risk. Polygenic risk score demonstrated superior performance over clinical scores for breast and prostate cancer, while the combined model outperformed the other 2 scores. For breast cancer (**A**), the cumulative incidence in the highest quantile of the combined score was 13 times greater than that in the lowest quantile. The difference was even more pronounced for prostate cancer (**B**), where participants in the highest combined score quantile showed 88-fold higher risk than the lowest quantile.

Furthermore, we divided the risk scores (polygenic risk scores, clinical factors, and the linear combined score through Cox proportional hazards models) into 11 quantiles (<10%, 10%-20%, 20%-30%, 30%-40%, 40%-50%, 50%-60%, 60%-70%, 70%-80%, 80-95%, and >95%) and calculated the quantile-specific cumulative incidence of cancer types. The developed polygenic risk scores showed a great ability to discern high-risk participants for breast and prostate cancers. The cumulative incidence in the highest quantile of polygenic risk scores was nearly 9 and 11 times the value in the lowest quantile for breast cancer (10.9% vs 1.3%) and prostate cancer (14.3% vs 1.3%), respectively. Additionally, the polygenic risk scores showed a better ability to stratify the high-risk population compared with clinical scores for breast and prostate cancers (Figure 4). The cumulative incidence in the highest quantile was higher for polygenic risk scores than for clinical risk scores (10.9% vs 10.1% for breast cancer and 14.3% vs 13.2% for prostate cancer) and lower in the lowest quantile for breast cancer (1.3% vs 2.2% for breast cancer). Moreover, the combined score incorporating polygenic risk scores and clinical risk factors could further improve the risk stratification, where the risk for breast cancer in the highest quantile of the combined score was 13 times the risk in the lowest quantile (13.7% vs 1.1%); for prostate cancer, it was 88 times the risk in the lowest quantile (19.6% vs 0.2%). The combined model also showed the best stratification ability for other cancer types (Supplementary Figure 3, available online).

We also checked the associations between each variable and the risk for cancer in the combined model. Polygenic risk score was found to be a statistically significant predictor for all cancer types after adjusting for the effects of clinical factors, except for ovarian cancer, and the hazard ratios ranged from 1.17 (kidney cancer) to 1.95 (prostate cancer) (Supplementary Figure 4, available online). Most of the 8 cancer types showed statistically significant associations with the variable “Cancer diagnosed by doctor.” This variable (field identifier 2453) integrated all types of self-reported cancers at baseline visit. For each cancer of interest, we excluded cancers whose diagnosis date was earlier or equal to the recruitment date and thus kept incident cases only. Therefore, “Cancer diagnosed by doctor” represents previous diagnoses of other types of cancer. Smoking was identified as an important risk factor for bladder cancer, CRC, kidney cancer, and lung cancer. Other shared risk factors were age at recruitment, obesity-related traits, and immunity-related traits. Male patients showed a statistically significantly higher risk of bladder and CRC. Interestingly, diabetes-related traits, such as glycated hemoglobin were associated with an increased risk of bladder and pancreas cancer but a decreased risk of prostate cancer. An increased alcohol-intake frequency was found to be a statistically significant risk factor for breast cancer and CRC. Two known risk factors for breast cancer, the mother's history of breast cancer and hormone-replacement therapy, were also detected. For lung cancer, dental problems and lower education levels were associated with higher risk.

### Impact on age

**Figure 5.**
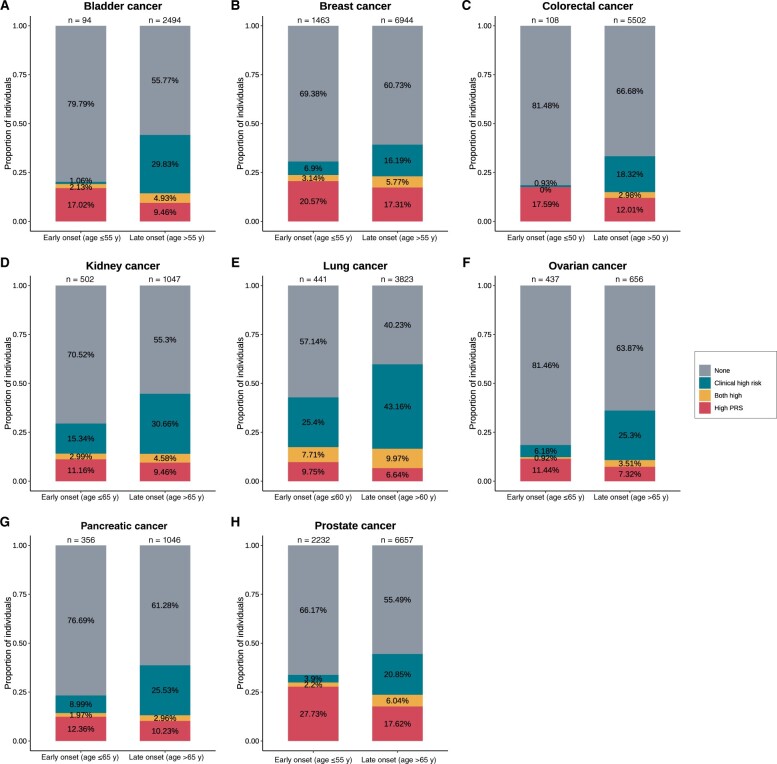
**Proportions of individuals with high polygenic risk scores and high clinical risk among those in the early-onset and late-onset groups across 8 cancer types**. We categorized patients with cancer into 2 groups based on the age of onset: an early-onset group and a late-onset group. Subsequently, we summarized the proportion of individuals with the top 10% polygenic risk scores (PRSs) and top 10% clinical risk scores. A similar pattern was observed across all 8 cancer types. The proportion of individuals with high PRSs was higher in the early-onset group than in the late-onset group, while the proportion of individuals with high clinical risk was higher in the late-onset group. Our results suggest that genetic risk factors tended to aggregate among early-onset patients, while late-onset patients tend to accumulate clinical risk factors.

The proportions of individuals with high polygenic risk scores and high clinical risk scores in the early-onset and late-onset groups are shown in Figure 5. The age cutoffs were 50 for CRC; 55 for bladder and breast cancers; 60 for lung cancer; and 65 for kidney, ovarian, pancreatic, and prostate cancers. Similar patterns were observed across 8 cancer types, where a larger proportion of individuals with high polygenic risk scores was identified in the early-onset group but a larger proportion of individuals with high clinical risk was found in the late-onset group. For breast cancer, the sample sizes of the early-onset and late-onset groups (1463 vs 6944) were more balanced than for other cancer types. Among early-onset cases (n = 1463), 20.6% had the top 10% polygenic risk scores, whereas in the late-onset group (n = 6944), the proportion was 17.3%. With regard to clinical risk, the proportions were 6.9% in the early-onset group; they were substantially higher, at 16.2%, in the late-onset group. Furthermore, we examined the associations between age of onset and polygenic risk score as well as clinical risk variables among patients with cancer through Cox proportional hazards models. No statistically significant associations were identified.

### Sensitivity analysis

The AUC values for polygenic risk score prediction generated from the logistic regression models were identical to the Cox proportional hazards model (Supplementary Table 7, available online). For clinical variable selection, the AUC values for the predetermined number of traits were comparable to those obtained from the Cox proportional hazards model, despite varying elbow points (Supplementary Figure 5, available online). Our results suggested no statistically significant differences between the logistic and Cox proportional hazards models for either polygenic risk score or clinical variable selection.

We compared the prediction accuracy and impact of age between the results generated from the entire sample (training + testing) and results generated by the testing samples. The AUC values of polygenic risk score and combined models were similar to the main results (Supplementary Figure 6, available online). For risk stratification, similar patterns, where a higher incidence rate of cancers in the highest quantile was found in polygenic risk scores compared with clinical factors, were identified for breast and prostate cancers. The combined model showed the best ability to stratify high-risk populations for all 8 cancer types (Supplementary Figure 7, available online). Finally, the proportions of high genetic and clinical risk between the early-onset and late-onset groups was calculated. Due to the extremely small sample size, the power of this analysis was limited, but for prostate cancer (which had the largest sample size here), the early-onset group had a larger proportion of individuals with high polygenic risk scores than did the late-onset group (14.7% vs 7.6%) and a lower proportion of individuals with high clinical risk scores than did the late-onset group (7.8% vs 9.2%), which was consistent with our main analyses.

Additionally, we assessed the calibration among testing samples; the results are shown in Supplementary Figure 8 (available online). Seven of the 8 cancer types showed a good calibration, with *P*-value larger than Bonferroni correction threshold (0.05/8 = 6.25 × 10^-3^) for goodness of fit test, while bladder cancer showed a statistically significant difference in predicted and observed absolute risk. For bladder cancer, there was an underestimated risk for high-risk individuals.

## Discussion

Through the analysis of data from the UK Biobank for 8 cancer types (bladder, breast, colorectal, kidney, lung, ovarian, pancreatic, and prostate), we constructed prediction models by incorporating the best-performed polygenic risk scores and clinical risk factors selected through XGBoost. The polygenic risk score accurately predicted risk for all 8 cancer types, and the performance showed a strong correlation with heritability, aligning with findings from previous studies (11-13). Furthermore, the inclusion of clinical variables improved the predictive performance, and the integrated model showed great prediction accuracy across the 8 cancer types and was able to identify high-risk individuals. Finally, we observed a relationship between the age of cancer onset and the risk components, where patients in the early-onset group had a higher genetic risk, while those in the late-onset group had higher clinical risk.

To our knowledge, this study is the first to employ XGBoost to select clinical risk factors and combine these variables with polygenic risk scores to predict risk of cancer. The incorporation of clinical variables statistically significantly enhanced model prediction accuracy, highlighting the importance of nongenetic factors in cancer development. Additionally, most of the selected clinical traits are known risk factors for cancer, reinforcing the validity of our model. For example, obesity-related and smoking-related traits, which have previously been reported to be cancer related (28-30), were consistently selected for all 8 cancer types in this study. The elevated concentration of glycated hemoglobin, as observed in our study, indicated an increased risk of bladder and pancreatic cancer and aligned with previous findings (31,32). Notably, our results corroborated previous research (33) by revealing a protective effect of type 2 diabetes and prostate cancer. Our study also identified alcohol intake frequency, a known risk factor for cancer (34,35), as a risk factor for breast cancer and CRC, which is consistent with the results from a meta-analysis. Furthermore, some cancer-specific risk factors were successfully identified in our study. For breast cancer, the history of breast cancer in the mother and the use of hormone-replacement therapy were statistically significant risk factors, a finding aligned with previous evidence (36,37). For lung cancer, loose teeth and dentures statistically significantly increased the cancer risk, supporting previous findings (38). Moreover, higher educational attainment was associated with a lower cancer risk, confirming the causal role (39). The evidence not only validates the results obtained through XGBoost but also highlights the biological plausibility and risk-prediction accuracy of the model developed by XGBoost.

Different models have been developed to assess the risk of various cancers. For example, 4 widely recognized models exist for breast cancer risk evaluation: IBIS, the Breast and Ovarian Analysis of Disease Incidence and Carrier Estimation Algorithm model, the Breast Cancer Risk Assessment Tool, and BRCAPRO. In a 10-year cohort study comparing the prediction performance of these models, IBIS showed superior performance (26). Therefore, we chose IBIS as a benchmark for comparison with our model. With 14 available variables in the UK Biobank (Supplementary Table 6, available online), the IBIS model achieved an AUC of 0.669 (95% CI = 0.655 to 0.682), which was 2.1% less than our model. This finding indicates the potential clinical applicability of our model for breast cancer. Moreover, our method can be effectively applied to any type or stage of cancer without the need for strong prior knowledge of cancer-associated clinical factors. In our study, we employed the same analysis pipeline for 8 different cancer types and generated meaningful results for each type, indicating that our model does not require strong prior knowledge of cancer-associated risk factors and can be generalizable to other cancer types or even other diseases.

Age of onset has long been recognized as an influential factor in the familial risk of breast cancer, and as early as 1990, a higher familial risk was found among patients with younger onset age (40). This observation provided a clue about the potential interaction between age of onset and genetic predisposition. The results of Mars et al. (13), however, contradicted previous findings, demonstrating a lower proportion of individuals with high polygenic risk scores in the early-onset group that could probably be attributed to the limited sample size. In our study, we identified a consistent pattern across 8 cancer types, where polygenic risk scores presented better prediction in patients in the early-onset group, while clinical risk was better calibrated for patients in the late-onset group. Our findings suggest practical implications for guiding cancer screening programs. A high polygenic risk score might suggest an early onset of cancer, while a high clinical risk might indicate a late onset of disease.

Several limitations in our study should be acknowledged, however. First, to address population stratification, our samples are restricted to individuals of European ancestry, which may hinder the generalizability of our results. Future studies should aim to include diverse ethnic groups for a more comprehensive analysis. Second, although logistic regression models were used in XGBoost, subsequent analyses used the Cox proportional hazards models, raising concerns regarding whether the traits XGBoost selected could accurately predict time to cancer. Although the results of the sensitivity analysis were not statistically significantly different from the main findings, for a more consistent analysis, future studies could consider using the same model. Third, due to the limited sample size of certain cancers, we combined the training and testing subsets for model fitting, which could lead to an overestimation of the prediction performance. Nonetheless, our sensitivity analysis indicated a minimal difference in prediction accuracy. Fourth, samples included in the GWAS summary statistics may also participate in the UK Biobank study, potentially leading to biased polygenic risk score construction. Methodologies checking and correcting such sample overlap should be included in future research. Finally, it is important to note that the clinical variables were measured during the baseline visit at recruitment, while past medical histories, such as self-report diseases and official medical diagnoses during follow-up, were ignored. To address this limitation, we intend to incorporate past medical history into our future studies.

Several important strengths should be noticed, as well. First, we used large-scale GWAS summary statistics and various methods for polygenic risk score derivation, thereby enhancing the robustness and precision of the developed polygenic risk score. Second, the implementation of XGBoost enabled us to select clinically relevant risk factors without any prior assumptions on risk factors, ensuring the generalizability of our model. Third, we conducted an extensive analysis of genetic and clinical risk factors for 8 cancer types using a large-scale dataset. Through the integration of polygenic risk score and pertinent clinical variables, we established models that accurately captured cancer risk.

This study has successfully developed cancer-specific polygenic risk scores and identified key clinical risk factors for 8 cancer types. Through the integration of genetic and clinical risk, we have constructed prediction models that achieved great accuracy across 8 cancer types. Additionally, we have observed an aggregate of genetic risk among patients with early-onset cancer and a predominance of clinical risk among patients with late-onset cancer. Notably, to our knowledge, this study is the first to use XGBoost to select clinical risk variables in the context of risk prediction. Our findings have demonstrated the effectiveness of XGBoost in variable selection and the potential for improving genetic risk prediction through the inclusion of clinical information. Our approach offers promising prospects for the establishment of robust risk-prediction models for the same patient groups across various cancer types.

## Supplementary Material

pkae008_Supplementary_Data

## Data Availability

The individual genotype and phenotype data underlying this article were provided by the UK Biobank by permission (reference No. 29900). The instructions to apply for the data can be found at https://www.ukbiobank.ac.uk/enable-your-research/apply-for-access. The GWAS summary statistics were downloaded from publicly available databases, and the information about related articles is available in Supplementary Table 2 (available online). The summary-level data (eg, polygenic risk score weights) will be available in the Polygenic Score Catalog (https://www.pgscatalog.org/), when published.
