# Supplementary material for: Using clinical and genetic risk factors for risk prediction of 8 cancers in the UK Biobank
Source: JNCI Cancer Spectr. 2024 Feb 14;8(2):pkae008. doi: 10.1093/jncics/pkae008 (PMC10919929; doi:10.1093/jncics/pkae008)
Supplement: pkae008_Supplementary_Data [file pkae008_supplementary_data.zip › Supplementary Figures.pdf]

Supplementary Materials

Supplementary Figure 1. Flowchart of data analysis

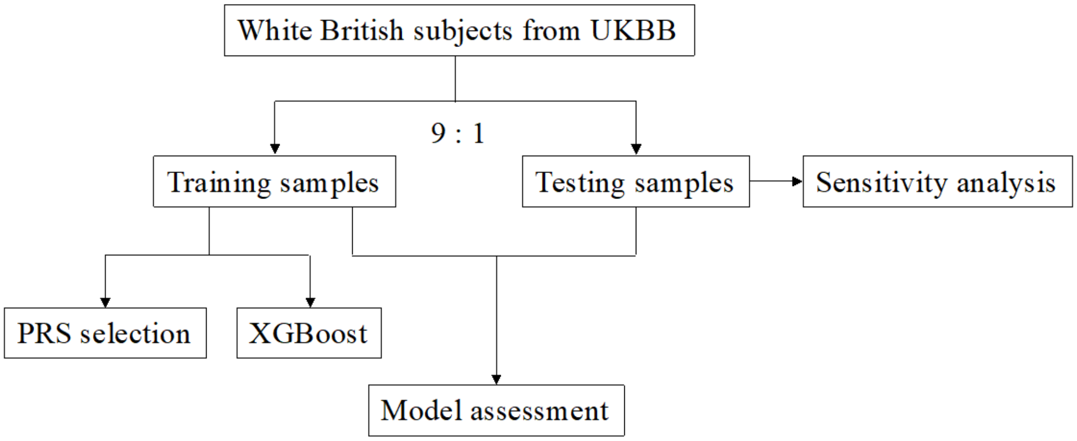

We split the White British subjects in the UKBB into a 9 to 1 training and testing sets. The training samples were used for PRS and clinical variables selection via five-fold cross validation. We then combined the training and testing samples to develop integrated prediction models and evaluated the performance. Sensitivity analysis assessing the potential overfitting problem was conducted using testing samples.

Supplementary Figure 2. Correlations between traits and PRS for seven cancers (bladder, colorectal, kidney, lung, ovarian, pancreas, and prostate cancers)

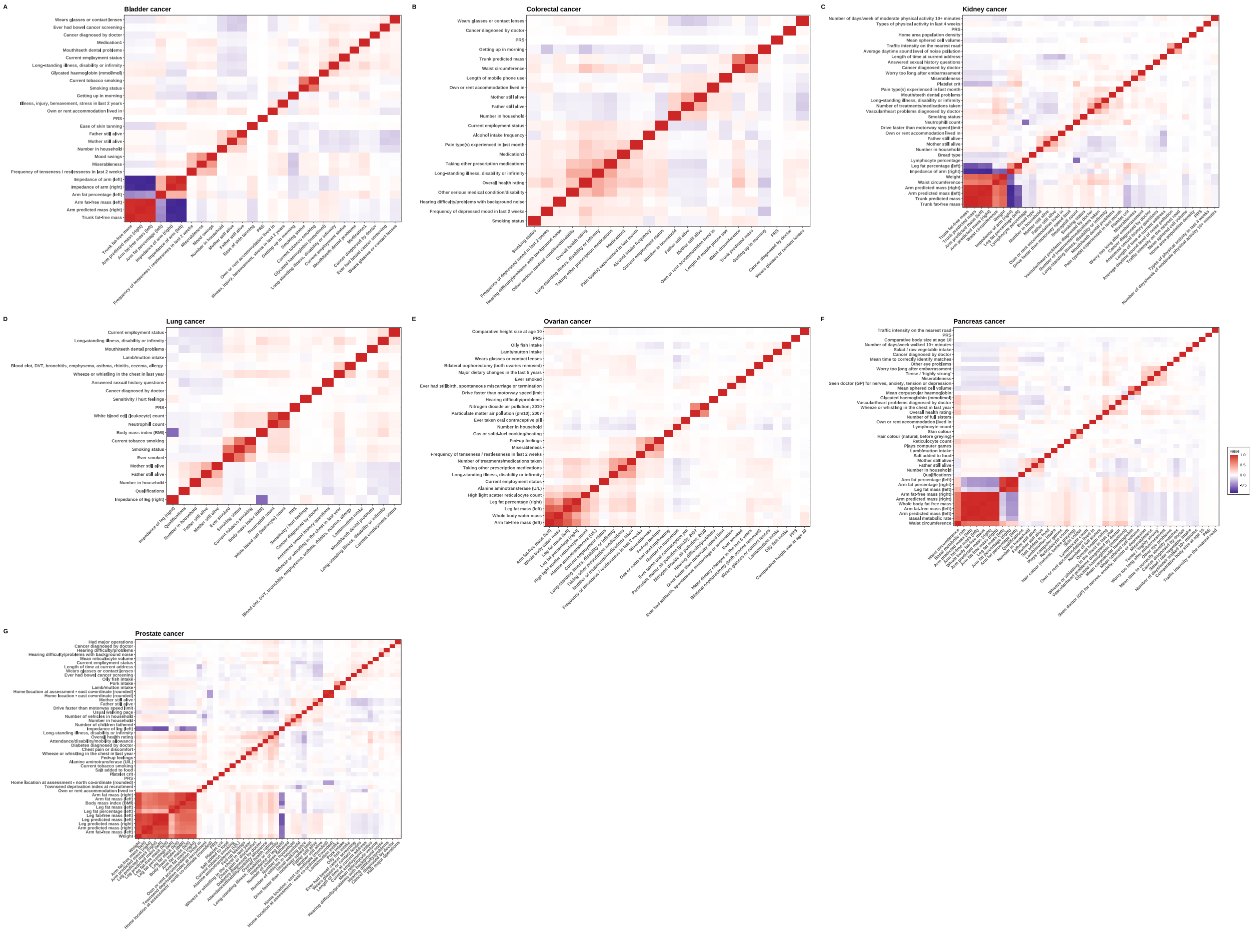

Correlations among variables selected for each cancer. Medication1: medications for pain relief, constipation, heartburn; medication2: for cholesterol, blood pressure, diabetes, or take exogenous hormones. We observed similar patterns across eight cancers. PRS was slightly correlated with clinical variables, and clinical variables showed several shared clusters such as obesity-related cluster and health condition-related cluster.

Supplementary Figure 3. Cumulative incidence of cancer in quantiles of three cancer risk scores

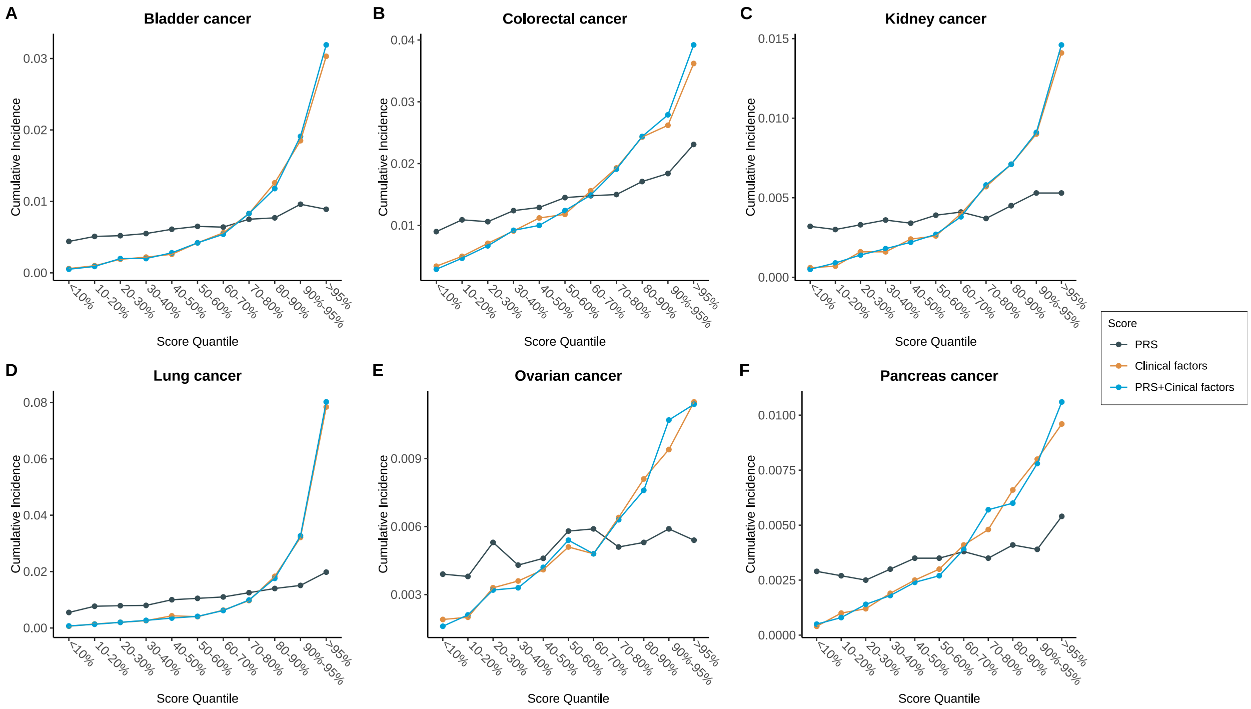

We divided PRS, clinical risk score, and the combined risk score into 11 quantiles and counted the cumulative incidence within each quantile. For the six cancers above, clinical risk score was superior to PRS in stratifying high- and low-risk individuals, and the combined score showed slight improvement to the clinical risk score.

Supplementary Figure 4. Hazard ratios for variables significantly associated with cancer risk

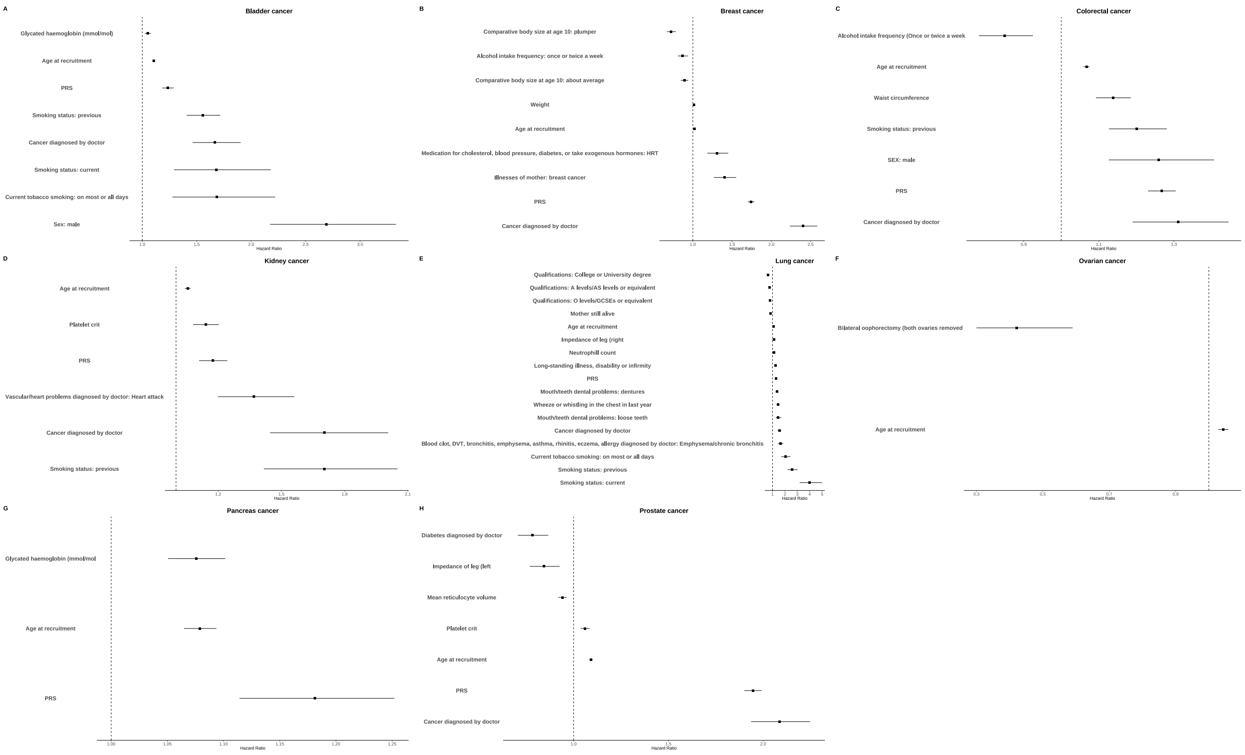

We fitted models for cancer with PRS and clinical variables and present the hazard ratios (HRs) for variables that were significant after Bonferroni correction. PRS was significantly associated with seven cancers except for ovarian cancer and the HR ranged from 1.17 for kidney cancer to 1.95 for prostate cancer. Significant clinical risk factors that were shared by at least two cancers included obesity-related traits, smoking, previous diagnosis of cancer, age at recruitment, immunity-related traits, and diabetes-related. Cancer-specific risk factors were observed for breast cancer (the mother's history of breast cancer and hormone-replacement therapy (HRT)) and lung cancer (dental problems and lower education levels).

Supplementary Figure 5. AUCs for clinical variable selection through logistic regression

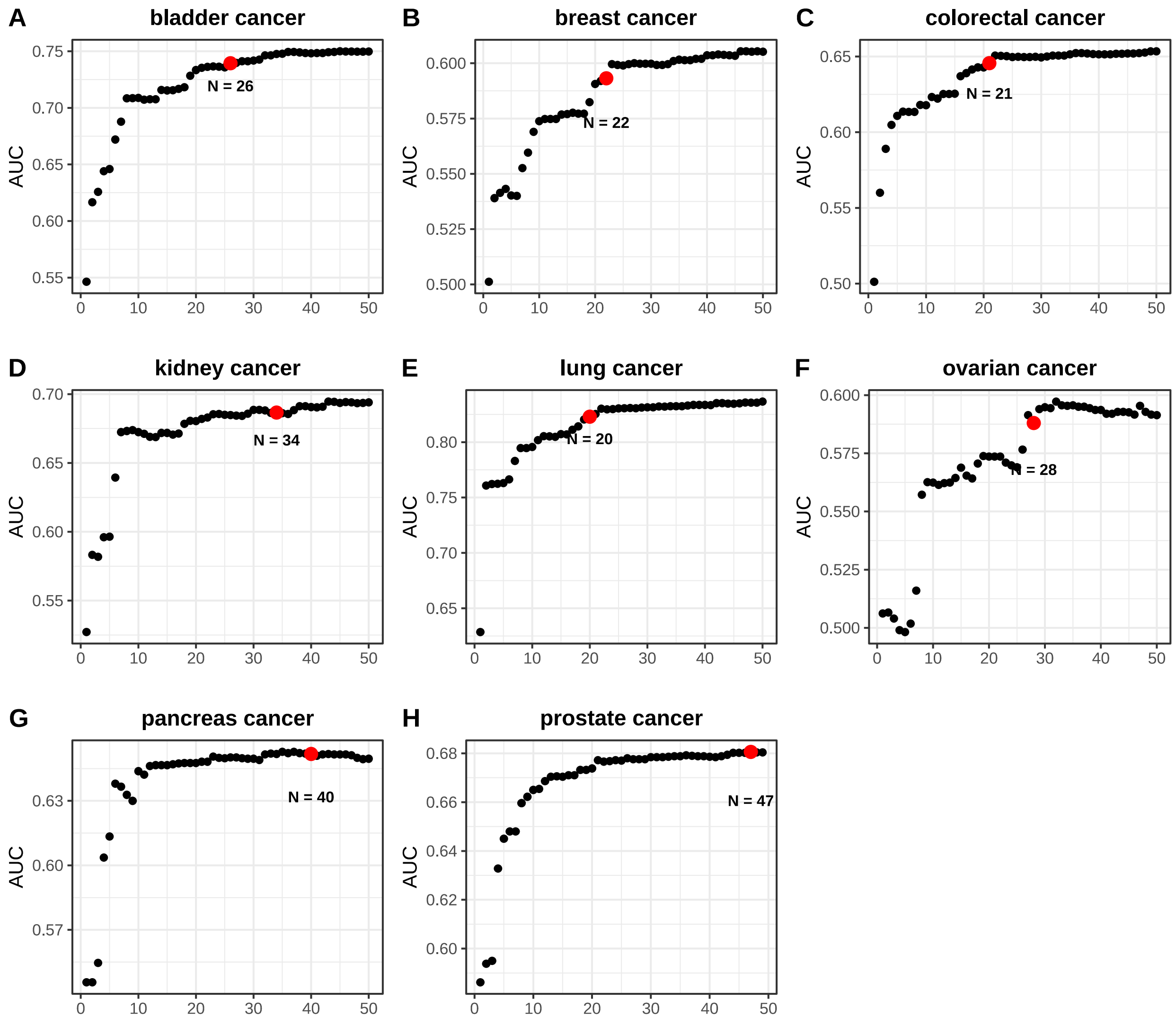

To assess the impact of inconsistent models, we re-ran the clinical variable selection through the logistic regression model. The AUC of each model against the number of clinical traits included is shown above. The elbow point is highlighted, which was decided by the Cox proportional hazard model in our main analysis. The logistic regression model exhibited similar AUCs to the Cox proportional hazard model for the elbow, suggesting negligible impact of the model inconsistency.

Supplementary Figure 6. ROC curves among testing samples

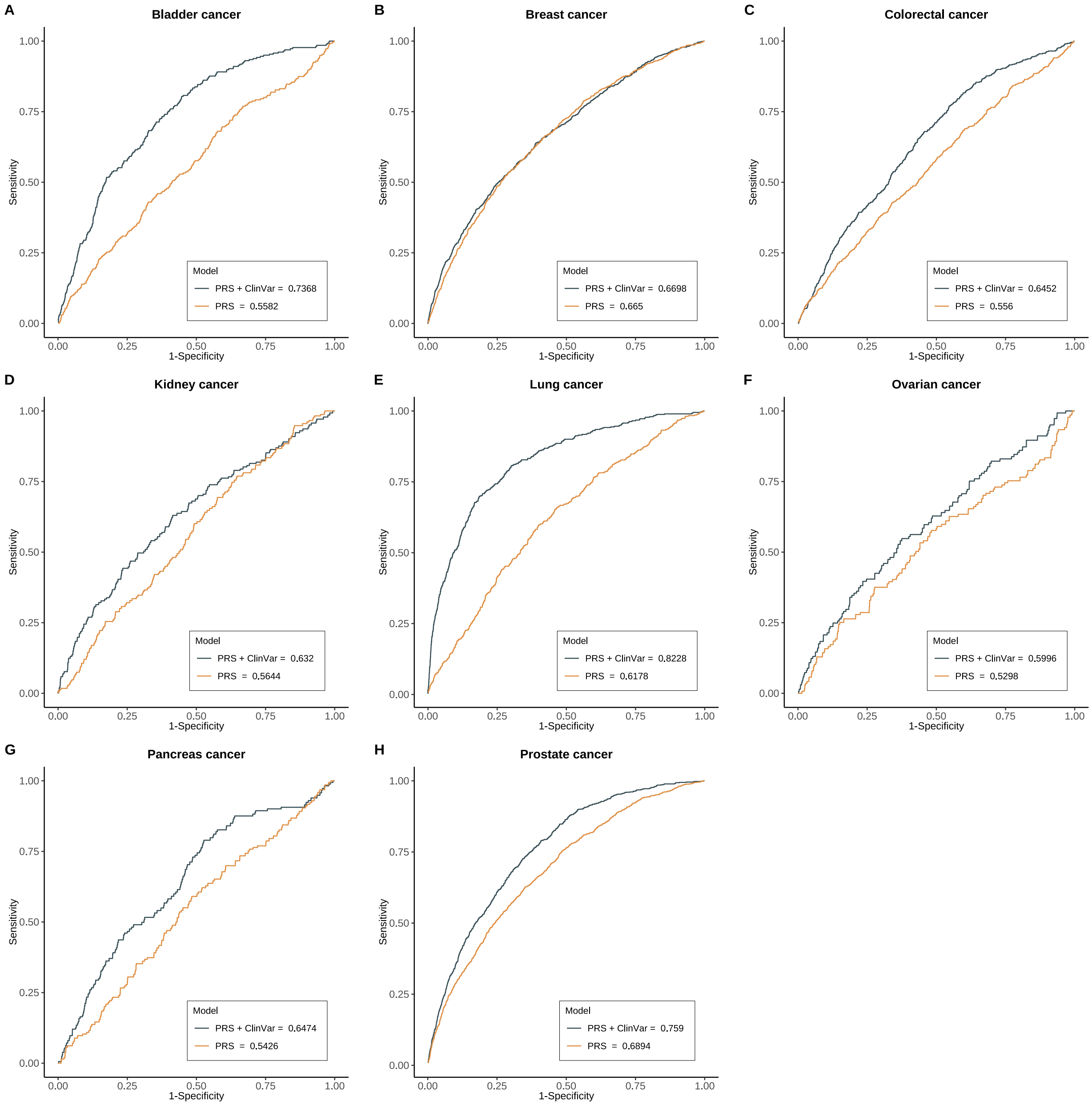

We assessed possibility of overfitting through assessment of the prediction performance among testing samples. The AUCs for PRS in testing samples were similar to the ones in combined samples, and the AUCs for the integration of PRS and clinical variables declined slightly in testing samples. The overall accurate prediction was maintained in testing samples.

Supplementary Figure 7. Quantile-specific cumulative incidence in testing samples

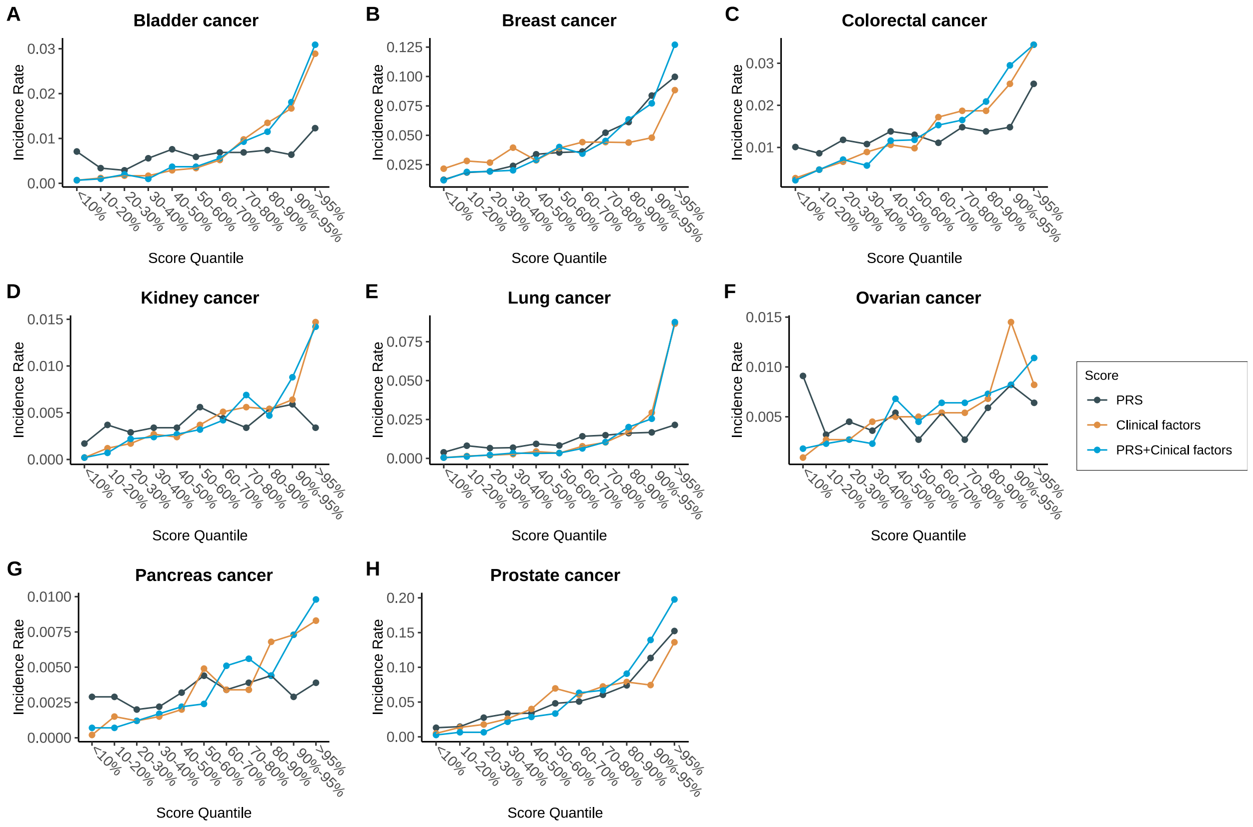

Among testing samples, we divided the PRS, clinical factor risk score, and the combined score into 11 quantiles, and the cumulative incidence of cancer was calculated in each quantile. For most cancers the integrated model outperformed the other two scores in stratifying high-risk individuals from the low-risk individuals, similar to our main findings.

Supplementary Figure 8. Calibration plots in testing samples

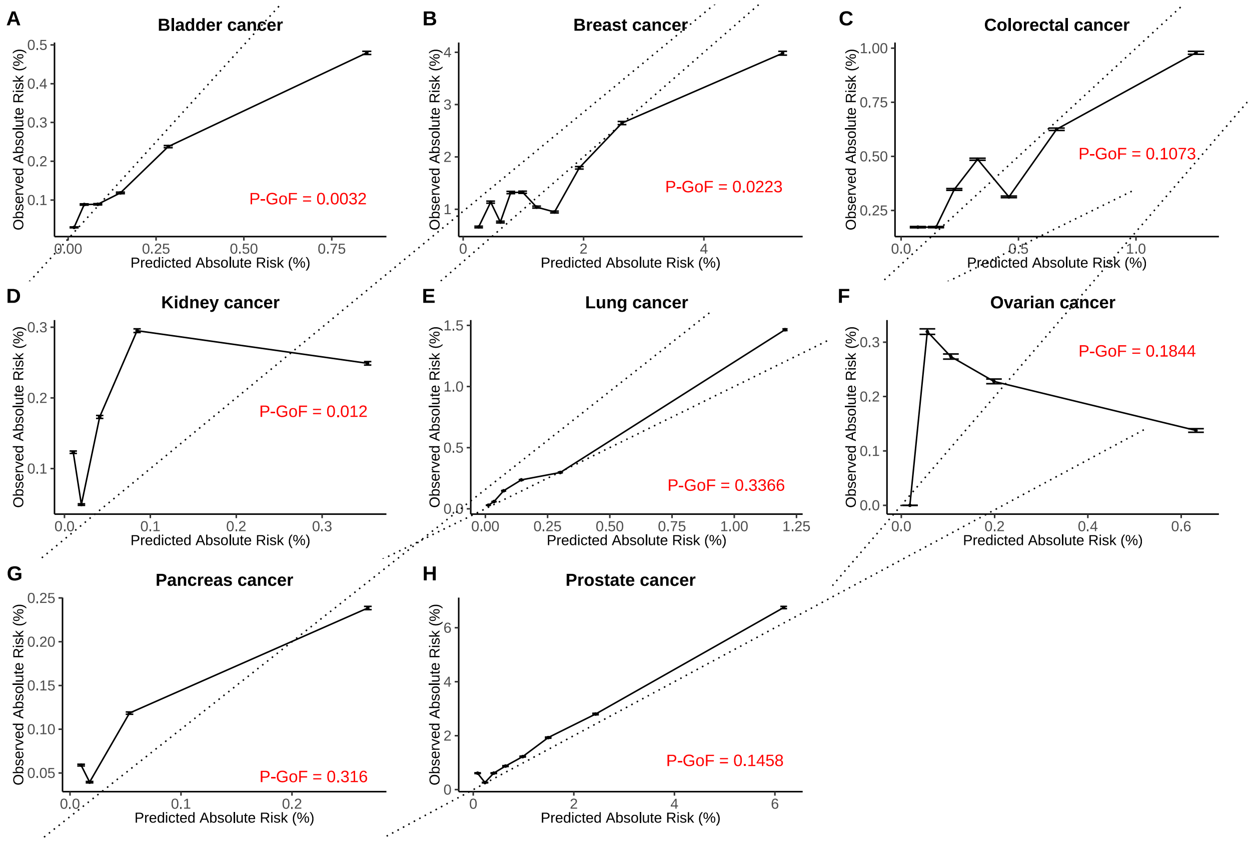

We evaluated the model calibration by plotting the predicted 5-year survival (x-axis) versus the observed one (y-axis) with appropriate quantiles. The Hosmer-Lemeshow goodness-of-fit statistic was calculated and tested. The p-values re shown in red (P-GoF). Seven of the eight cancers showed a good calibration with P-GoF>0.05/8 while the bladder cancer showed a significant difference in predicted and observed absolute risk.
